# Intramuscular quadratus lumborum block can be a good analgesic option for lumbar spine surgery

**DOI:** 10.1186/s40981-024-00758-5

**Published:** 2024-12-20

**Authors:** Keisuke Yoshida, Shiori Tanaka, Takayuki Hasegawa, Tatsumi Yakushiji, Satoki Inoue

**Affiliations:** 1 Department of Anesthesiology, Fukushima Medical University School of Medicine, Fukushima, Fukushima Japan; 2 Department of Anesthesiology, Aidu Chuo Hospital, Aizuwakamatsu, Fukushima Japan

To the Editor,

For pain management after lumbar spine surgery, several regional anesthetic techniques, such as thoracolumbar interfascial plane block, erector spinae plane block, and wound infiltration anesthesia, have been investigated [1–3]. However, none of these have become widely used and are not recommended in spine surgery [4]. Here, we report a case of successful postoperative pain management with intramuscular quadratus lumborum block (QLBi) in a 65-year-old male patient (168 cm, 97 kg). He underwent L4/5 laminectomy under general anesthesia with propofol, remifentanil, ketamine (30 mg at the beginning of the surgery, followed by 7.6 mg/h until the end of the surgery, at a total of 45 mg), and rocuronium. Intraoperatively, flurbiprofen axetil 50 mg was administered, followed by bilateral ultrasound-guided QLBi with 30 mL (per side) of 0.25% ropivacaine in the prone position. He needed only oral celecoxib 100 mg within the first 24 h postoperatively with Numerical Rating Scale pain scores 0–1, and had no complications.

One of the strengths of quadratus lumborum block (QLB) is that it does not increase the risk of surgical site infection (SSI) because it does not have to be performed close to the surgical wound. Thus, QLBi is more preferable in spinal surgery because treatment of SSI is not easy should it occur. In addition, anterior QLB is a relatively difficult technique although its effectiveness has been reported in lumbar spine surgery [5]; thus, we have focused on QLBi, which is technically easier and safer to perform. Moreover, QLBi is expected to be more effective on the dorsal branches of the spinal nerves and to hardly affect the ventral branches because the injection site is located more dorsally. Although the mechanism of QLBi and the spread of the local anesthetics have not been elucidated at the time of writing, the risk of postoperative muscle weakness of the lower limbs related to QLBi is a concern. However, in our previous study on QLBi for cesarean section [6], we did not observe any motor impairments that could have been related to QLBi. In the present study, there is a possibility that early motor deficits might be masked because QLBi was performed with spinal anesthesia. Therefore, we believe that QLBi may be a good analgesic option after lumber spine surgery, and we are currently conducting a randomized controlled trial to investigate its effects after lumbar spine surgery with instrumentation, which is associated with significant postoperative pain (clinical trial registry: jRCTs021240026).

## Data Availability

Not applicable.
